# Patient-reported experiences during and following treatment with belantamab mafodotin for relapsed/refractory multiple myeloma in the DREAMM-2 study

**DOI:** 10.3389/fonc.2023.1274659

**Published:** 2023-12-07

**Authors:** Anna Cardellino, Julia R. Correll, Mona Martin, Boris Gorsh, Sandhya Sapra, Rakesh Popat

**Affiliations:** ^1^Patient Centered Outcomes, GSK, Upper Providence, PA, United States; ^2^Patient-Centered Research, Evidera, Seattle, WA, United States; ^3^NIHR/University College London Hospital Clinical Research Facility, NHS Foundation Trust, London, United Kingdom

**Keywords:** belantamab mafodotin, multiple myeloma, embedded qualitative interviews, disease impact, B-Cell maturation antigen, immunomodulatory drug

## Abstract

**Introduction:**

Patients with relapsed or refractory multiple myeloma (RRMM) are likely to be living with persistent symptoms, especially bone pain and fatigue, and experiencing restrictions in their physical and social functioning, which reduce health-related quality of life.

**Methods:**

This qualitative interview study evaluated patients’ perspectives about living with RRMM and their treatment with belantamab mafodotin, using interviews embedded in the Phase II DREAMM-2 trial (NCT03525678) with belantamab mafodotin. Patients consented to participate in up to 2 recorded telephone interviews (at treatment cycle 4 [C4] and at end of treatment [EOT]) comprising open-ended questions.

**Results:**

A total of 142 interviews were conducted with 111 unique patients. At C4, common symptoms included neuropathy, fatigue, and bone or joint pain. Improvements in symptom severity were reported by patients who responded to belantamab mafodotin. Symptoms associated with visual impairment, eye irritation, and eye pain reported during the trial were reported to be at- or near-resolution by the EOT interview. Regarding impacts of underlying MM, patients most commonly expressed concerns about changes in daily performance and lifestyle for both responders (67.5% of all impact expressions) and non-responders (63.2%). Overall, interview participants reported being satisfied with belantamab mafodotin treatment.

**Discussion:**

This qualitative patient interview study provides valuable insight into patients’ symptomatic experience with belantamab mafodotin for their RRMM treatment and may help healthcare providers better anticipate their patients’ real-world experience and needs when prescribing this novel agent in the clinic.

## 1 Introduction

The incidence of multiple myeloma (MM) is highly variable across countries but has increased uniformly since 1990 (1, 2). This hematologic cancer disproportionately affects older patients (median age of diagnosis >70 years) in whom the burden of adverse events and decline in health-related quality of life (HRQoL) can be particularly high (3, 4).

Although there have been many therapeutic advances over the past 20 years, including in older patients, MM remains a challenging and incurable disease, with almost all patients experiencing relapse and eventually becoming refractory to available therapies (5–7). Treatment for relapsed or refractory MM (RRMM) involves several lines of therapy and the time between treatments typically shortens with each subsequent line (5, 7). Due to the shorter amount of time spent in remission, patients with RRMM are likely to be living with persistent symptoms, especially bone pain and fatigue, and experience restrictions in their physical and social functioning, all of which reduce overall HRQoL (8–11). Treatment-related adverse events also add to this disease burden, highlighting the importance of considering the adverse event profile of regimens when selecting treatment for these patients (11).

HRQoL is highly relevant for patients with RRMM and thus should be closely assessed alongside metrics such as treatment response rate and survival (3, 9, 12). Indeed, many Phase III clinical trials in MM incorporate HRQoL as an endpoint, although typically as a secondary outcome (4). In addition to efficacy and safety results, there has been growing interest in incorporating patient-reported outcomes data to guide evidence-based decision-making and treatment selection in patients with RRMM to ensure that their HRQoL is maintained or improved (10, 13). However, qualitative data from patient interviews on the impact of disease- and treatment-related symptoms, especially among patients with triple-class refractory RRMM, are limited (11, 14, 15). Open-ended interviews with patients enrolled in therapeutic clinical trials can provide valuable insights to complement understanding of a treatment’s safety profile; for example, providing a greater understanding of the timing and severity of side effects. This patient experience can also be used to improve future study design and clinical trial implementation.

Belantamab mafodotin is a first-in-class B-cell maturation antigen (BCMA)-targeted monoclonal antibody conjugated to the microtubule-disrupting agent monomethyl auristatin F (MMAF) (16). In the pivotal, open-label DREAMM-2 study (NCT03525678), belantamab mafodotin monotherapy demonstrated rapid, deep, and durable responses, with a manageable safety profile in patients with triple-class refractory RRMM with 13 months follow-up (17).

The objective of this qualitative interview study was to understand patients’ perspectives of living with RRMM and their treatment with belantamab mafodotin in the DREAMM-2 trial.

## 2 Methods

### 2.1 Study design

This qualitative interview study (GSK study 205678) collected data from individual telephone interviews with a sub-sample of patients who received treatment in the DREAMM-2 clinical study.

Institutional review board and ethics committee approval was received for this interview-based study as an amendment to the DREAMM-2 clinical trial protocol. Informed consent for audio recording of the interview was provided by patients as part of their agreement to participate. This study was performed in accordance with the ethical principles that have their origin in the Declaration of Helsinki and that are consistent with good clinical practice and applicable regulatory requirements and complied with all applicable laws regarding patient privacy. After conclusion of each interview, contact information was destroyed by the interviewer.

### 2.2 Study population

The DREAMM-2 study qualitative interviews took place between 2018 and 2022 and included patients from 40 clinical sites in 8 countries: Australia, Canada, France, Germany, Italy, Spain, the United Kingdom, and the United States. All patients met the clinical trial eligibility criteria for DREAMM-2 (17, 18), including having RRMM disease progression following 3 or more prior lines of therapy. Patients consented to participate in up to 2 recorded telephone interviews as part of the clinical trial protocol. Patients from all 3 belantamab mafodotin treatment groups were eligible to participate. Responders were defined as those with complete or partial responses per International Myeloma Working Group (IMWG) Response Criteria by Independent Review Committee (IRC). Responders were able to stay on belantamab mafodotin until disease progression, unacceptable toxicity, withdrawal of consent, death, or end of study, whichever came first. Patients who achieved complete response, had completed at least 8 cycles of therapy, and maintained CR for at least 2 additional treatment cycles could also discontinue treatment and enter the follow-up period.

### 2.3 Data collection

Patients were invited to participate in interviews at treatment cycle 4 (C4) and at end of treatment (EOT). Interviews were conducted within 21 days following C4 and EOT. Patients discontinuing treatment before C4 and those who did not partake in a C4 interview were also eligible for an EOT interview. Patients interviewed at C4 proceeded to EOT interviews unless their EOT visit was within 30 days of their C4 interviews.

Telephone interviews were conducted in the patient’s native language (English, Spanish, French, German, or Italian). A total of 10 interviewers were trained and monitored by Evidera, a third-party vendor. All interviewers were experienced in qualitative research and additional study specific training was provided, including adverse event reporting and RRMM-specific information.

During the interviews, patients were asked open-ended questions on their experience of symptoms, impacts, and treatment during the study. Patient-perceived symptom severity was rated on a 0–10 numerical rating scale (NRS; 0=not severe; 10=extremely severe). Patients were also asked to rate their degree of satisfaction with study treatment on a 0–10 NRS, with 0 defined as “not at all satisfied” and 10 as “extremely satisfied.”

All interviews were audio-recorded and transcribed for analysis. English-language audio files were developed from non-English interviews with assistance from simultaneous interpreters. All transcripts were quality checked against the original interview audio file for completeness and accuracy, and any identifying information was removed.

### 2.4 Analytical methods

A mixed methods approach was used to combine qualitative data from the conversational aspects of the interviews with quantitative data from the rating exercises and from selected variables in the clinical trial dataset, including responder status. A “responder” was any patient with a partial response or better, confirmed by an independent review committee (17).

Qualitative data were coded and organized by thematically similar content. A preliminary set of codes was formulated based on study objectives and the questions asked in the interview guide. The dictionary of codes was then expanded as subsequent interviews were coded. The study assessed the prevalence of continuing symptoms (started before and persisted during the trial) and new symptoms (started during the trial), while symptoms that resolved prior to participation in the trial were not coded. This article reports data on continuing symptoms only, which are referred to throughout the article as “disease symptoms.” Similarly, continuing impacts that commenced prior to the study and continued throughout its duration are referred to as “impacts.” Ocular adverse events are a known class effect of MMAF-containing anti-drug conjugates and were observed in patients receiving belantamab mafodotin in clinical studies (19, 20). Because of this, ocular adverse events were explored in depth during patient interviews and are summarized separately from other symptoms.

Transcripts from the initial (C4) interviews were used to assess saturation of concept and were organized sequentially and grouped into 10 groups of 11 or 12 transcripts per group. Each transcript group was compared with the previous groups to identify new concepts using newly established concept codes, and to assess concept saturation (the point at which no new concepts are identified, suggesting that a sufficient number of interviews have been conducted). Five interview transcripts were selected at random, independently dual coded using 2 separate coders, and compared for inter-coder agreement in code assignment.

## 3 Results

### 3.1 Patient characteristics

Of the 221 patients enrolled in the DREAMM-2 study (18, 21), 111 patients participated in qualitative interviews. The demographic and clinical characteristics of patients interviewed are described in **Table 1**; 50 (45.0%) were female, the median (range) age was 66.0 (40–89) years, and the majority of patients were White (n=88 [79.3%]). Patients had completed a median (range) of 6 (3–21) prior lines of therapy. The median time from initial diagnosis of MM to screening for inclusion in DREAMM-2 was 5.9 (1.1–12.1) years, and the median time on study treatment was 26.9 (3–186) weeks. The overall response rate at C4 interview was 56.7% (**Table 1**). C4 interviews were conducted at a median of 105 days (~3.5 months) after C1D1.

**Table 1:** Demographics and clinical characteristics of interviewees.

	C4 interview (n=104)	EOT interview (n=38)	Total (N=111)
Gender, n (%)Female Male	48 (46.2) 56 (53.8)	18 (47.4) 20 (52.6)	50 (45.0) 61 (55.0)
**Age at consent to interview, years, median (range)**	66.0 (40–89)	63.5 (46–89)	66.0 (40–89)
**Time since first diagnosed with MM, years, median (range)**	6.0 (1.1–12.1)	6.0 (4.1–12.1)	5.9 (1.1–12.1)
**Prior lines of therapy, median (range)**	6.0 (3–21)	5.5 (3–21)	6.0 (3–21)
**Time on study treatment, weeks, median (range)**	24.9 (3–186)	54.6 (12–186)	26.9 (3–186)
ORR*, n (%)	59 (56.7)	31 (81.6)	63 (56.8)
Country of residence, n (%)			
United States	69 (66.3)	25 (65.8)	72 (64.9)
Spain	7 (6.7)	5 (13.2)	10 (9.0)
France	8 (7.7)	2 (5.3)	9 (8.1)
United Kingdom	7 (6.7)	2 (5.3)	7 (6.3)
Germany	6 (5.8)	2 (5.3)	6 (5.4)
Canada	5 (4.8)	1 (2.6)	5 (4.5)
Australia	1 (1.0)	1 (2.6)	1 (0.9)
Italy	1 (1.0)	0	1 (0.9)
Ethnicity, n (%)			
Black or African American	17 (16.3)	7 (18.4)	18 (16.2)
Asian	3 (2.9)	1 (2.6)	3 (2.7)
White	82 (78.8)	30 (78.9)	88 (79.3)
Multiple	2 (1.9)	0	2 (1.8)

*Overall response rate is the sum of stringent complete responses, complete responses, very good partial responses, and partial responses.

C4, cycle 4; EOT, end of treatment; MM, multiple myeloma; ORR, overall response rate.

### 3.2 Interview participants

Of the 111 patients interviewed, 104 completed initial interviews before or at C4, including 33 patients who discontinued the trial early without reaching C4. Both a C4 and an EOT interview were completed for 31 patients. An additional 7 patients completed an EOT interview only.

In the C4 interview subgroup, 58 (55.8%) patients were identified as responders to belantamab mafodotin treatment; 31 (81.6%) patients in the EOT interview were classified as responders.

### 3.3 Inter-coder agreement

Inter-coder agreement, representing consistency between coders, was high, ranging from 91.3% to 98.1% for the code assigned in the 5 dual coded transcripts. These results exceeded the suggested threshold of 90% agreement (22).

### 3.4 Saturation of concept

Overall, 149 individual symptom and impact concepts were expressed by patients during their C4 interviews. While there was a gradual decrease in the emergence of new concepts with each subsequent transcript group, 8 new concepts (5%) arose in the final transcript group: 4 ocular symptoms (depth perception changes, distorted vision, peripheral vision affected, and starry vision) and 4 additional symptoms (circulation problems, septic shock, throat itching, and throat soreness). A cutoff of ≤5% new information has been suggested to meet saturation for exploratory qualitative research (23).

### 3.5 Disease symptoms

At the C4 interviews, the most commonly reported symptoms among treatment responders (n=58) were neuropathy/numbness (67%), fatigue (53%), tiredness (41%), bone/joint pain (41%), and back pain (38%) (**Table 2**). At the start of the study, responders provided the highest (worst) symptom severity ratings (on a NRS of 0–10) for bone pain (6.9), numbness (6.0), bone fractures (5.8), and constipation (5.8) (**Table 3**). By the time of C4 interview, the severity ratings for these symptoms had reduced to 3.6, 5.8, 3.3, and 3.7, respectively.

**Table 2:** Number of patients reporting symptoms at C4 and EOT interviews.

	C4 interviews		EOT interviews	
Symptoms	Responders (n=58)	Non-responders (n=46)	Responders (n=31)	Non-responders (n=7)
Energy-related symptoms				
Fatigue	31 (53)	23 (50)	13 (42)	0
Tiredness	24 (41)	24 (52)	15 (48)	3 (43)
Weakness	18 (31)	15 (33)	6 (19)	1 (14)
Low energy	7 (12)	5 (11)	2 (6)	1 (14)
Pain & discomfort symptoms				
Bone or joint pain	24 (41)	27 (59)	8 (26)	4 (57)
Back pain	22 (38)	17 (37)	9 (29)	1 (14)
Muscle weakness	13 (22)	8 (17)	3 (10)	1 (14)
Bone symptoms				
Bone fractures	6 (10)	4 (9)	2 (6)	0
Respiratory symptoms				
Shortness of breath	19 (33)	16 (35)	10 (32)	1 (14)
Respiratory infections	6 (10)	5 (11)	2 (6)	0
Digestive symptoms				
Loss of appetite	15 (26)	8 (17)	4 (13)	1 (14)
Constipation	12 (21)	8 (17)	5 (16)	1 (14)
Diarrhea	8 (14)	4 (9)	4 (13)	1 (14)
Urinary symptoms				
Frequent urination	13 (22)	7 (15)	2 (6)	3 (43)
Extreme thirst	6 (10)	4 (9)	2 (6)	0
Systemic symptoms				
Dizziness	6 (10)	5 (11)	3 (10)	0
Ocular symptoms				
Cataracts	6 (10)	1 (2)	3 (10)	0
Additional symptoms				
Neuropathy or numbness	39 (67)	24 (52)	16 (52)	2 (29)
Bruising or bleeding easily	19 (33)	9 (20)	5 (16)	0
Swelling	13 (22)	6 (13)	4 (13)	1 (14)
Itching	10 (17)	2 (4)	6 (19)	1 (14)

Symptoms reported by ≥10% of patients presented.

C4, cycle 4; EOT, end of treatment.

**Table 3:** Severity ratings* for the most commonly rated disease symptoms and all ocular symptoms at C4 interviews^†^ (n=104).

Symptom	Responders^‡^ (n=58)			Non-Responders (n=46)		
	Patients rating symptom, n (%)	Severity rating at start of study, mean (SD)	Severity rating by C4 interview, mean (SD)	Patients rating symptom, n (%)	Severity rating at start of study, mean (SD)	Severity rating by C4 interview, mean (SD)
**Fatigue**	41 (70.7)	4.6 (2.5)	3.4 (2.4)	27 (58.7)	4.4 (2.1)	4.5 (2.4)
**Neuropathy**	24 (41.4)	4.5 (2.6)	3.7 (2.5)	9 (19.6)	3.9 (1.9)	2.8 (2.0)
**Back pain**	18 (31.0)	5.2 (2.5)	4.6 (2.9)	13 (28.3)	4.7 (2.7)	4.2 (2.7)
**Weakness**	14 (24.1)	4.4 (2.0)	4.1 (2.9)	9 (19.6)	5.4 (2.1)	4.8 (1.8)
**Bone pain**	12 (20.7)	6.9 (2.1)	3.6 (3.1)	9 (19.6)	4.9 (2.5)	4.4 (2.3)
**Bleeding or bruising easily**	11 (19.0)	5.2 (2.7)	2.7 (2.8)	6 (13.0)	2.8 (1.2)	2.6 (1.5)
**Pain**	9 (15.5)	5.4 (2.7)	3.4 (2.8)	4 (8.7)	8.0 (2.2)	4.3 (3.2)
**Shortness of breath**	9 (15.5)	4.0 (1.9)	2.7 (2.9)	7 (15.2)	4.1 (2.3)	4.1 (3.0)
**Loss of appetite**	7 (12.1)	5.0 (3.3)	3.3 (2.9)	5 (10.9)	3.0 (1.0)	4.3 (1.7)
**Constipation**	6 (10.3)	5.8 (2.2)	3.7 (2.3)	7 (15.2)	3.6 (1.9)	1.5 (1.8)
**Numbness**	4 (6.9)	6.0 (1.8)	5.8 (3.6)	7 (15.2)	3.3 (2.1)	1.7 (1.2)
**Bone fractures**	4 (6.9)	5.8 (3.9)	3.3 (1.0)	6 (13.0)	4.3 (4.6)	3.3 (3.4)
Ocular symptoms						
Cataracts	1 (1.7)	6.0 (N/A)	–	0	–	–
Dry eyes	0	–	–	1 (2.2)	4 (N/A)	4 (N/A)
Ocular – eye hemorrhage	0	–	–	1 (2.2)	3 (N/A)	6 (N/A)
Ocular – dry eyes	1 (1.7)	3 (N/A)	3 (N/A)	1 (2.2)	5 (N/A)	4 (N/A)
Ocular – vision in prism	0	–	–	1 (2.2)	–	–

*Disease and treatment-related symptom severity was rated 0–10 (0=not severe; 10=most severe); ^†^Most common symptoms are those rated by ≥10% of patients; all rated ocular symptoms are presented, regardless of frequency; ^‡^Responders had ≥partial response by International Myeloma Working Group criteria.

C4, cycle 4; N/A, not applicable; SD, standard deviation.

At the C4 interviews, the most commonly reported symptoms among non-responders (n=46) were bone/joint pain (59%), neuropathy/numbness (52%), tiredness (52%), and fatigue (50%) (**Table 2**). At the start of the study, non-responders provided the highest symptom severity ratings for pain (8.0), weakness (5.4), bone pain (4.9), and back pain (4.7) (**Table 3**). By the time of C4 interview, the ratings for these symptoms had reduced to 4.3, 4.8, 4.4, and 4.2, respectively. Among the other commonly rated symptoms, the greatest improvements in symptom severity were for constipation (3.6 to 1.5) and numbness (3.3 to 1.7).

At the EOT interviews, the most commonly reported symptoms among responders (n=31) were neuropathy/numbness (52%), tiredness (48%), fatigue (42%), and shortness of breath (32%) (**Table 2**). Responders rated the worst symptom severity they experienced during the study for back pain (7.3), fatigue (6.7), and weakness (6.3) (**Table 4**). By the EOT interview, the reported severity of these symptoms had reduced to 2.3, 3.5, and 5.5, respectively. Among the other commonly rated symptoms, the largest improvements in severity ratings were observed for swelling (decreased from 10.0 to 4.5) and loss of appetite (6.2 to 2.0).

**Table 4:** Severity ratings* for the most commonly rated disease symptoms and ocular symptoms at EOT interview^†^ (n=38).

Symptom^†^	Responders^‡^ (n=31)			Non-Responders (n=7)		
	Patients rating symptom, n (%)	Severity rating at its worst during study, mean (SD)	Severity rating by EOT interview, mean (SD)	Patients rating symptom, n (%)	Severity rating at its worst during study, mean (SD)	Severity rating by EOT interview, mean (SD)
**Fatigue**	17 (54.8)	6.7 (1.8)	3.5 (2.7)	5 (71.4)	5.2 (2.6)	3.8 (1.9)
**Neuropathy**	10 (32.3)	5.0 (2.8)	3.8 (2.9)	0	–	–
**Back pain**	7 (22.6)	7.3 (1.8)	2.3 (3.1)	0	–	–
**Shortness of breath**	7 (22.6)	5.4 (1.5)	1.2 (1.8)	1 (14.3)	9.0 (N/A)	8.0 (N/A)
**Weakness (generalized)**	5 (16.1)	6.3 (3.2)	5.5 (2.9)	2 (28.6)	5.5 (3.5)	4.5 (2.1)
**Loss of appetite**	5 (16.1)	6.2 (3.8)	2.0 (4.5)	2 (28.6)	4.5 (3.5)	4.5 (3.5)
**Anemia**	5 (16.1)	1.0 (1.0)	0.7 (1.2)	0	–	–
**Constipation**	5 (16.1)	3.0 (1.7)	2.0 (2.7)	0	–	–
**Bruising easily**	3 (9.7)	3.0 (4.4)	2.7 (4.6)	0	–	–
**Itching**	1 (3.2)	6.0 (N/A)	0 (N/A)	1 (14.3)	4.0 (N/A)	0 (N/A)
**Frequent urination**	0	–	–	2 (28.6)	5.5 (0.7)	3.5 (0.7)
**Pain (rib and shoulder)**	0	–	–	1 (14.3)	4.0 (N/A)	4.0 (N/A)
**Nausea**	0	–	–	1 (14.3)	6.0 (N/A)	0 (N/A)
Ocular symptoms						
Sensory change: vision	1 (3.0)	9 (N/A)	0 (N/A)	0	–	–

*Disease and treatment-related symptom severity was rated 0–10 (0=not severe; 10=most severe); ^†^Most common symptoms are those rated by ≥10% of patients; all rated ocular symptoms are presented; ^‡^Responders had ≥partial response by International Myeloma Working Group criteria.

EOT, end of treatment; N/A, not applicable; SD, standard deviation.

At the EOT interviews, the symptoms reported by ≥2 non-responders (n=7) were bone/joint pain (57%), tiredness (43%), frequent urination (43%), and neuropathy/numbness (29%) (**Table 2**). Non-responders rated the worst symptom severity they experienced during the study for shortness of breath (9.0), nausea (6.0), weakness (5.5), and frequent urination (5.5) (**Table 4**). By the time of EOT interview, the reported severity of these symptoms had reduced to 8.0, 0, 4.5, and 3.5, respectively.

#### 3.5.1 Impacts

At the C4 interviews, nearly two-thirds of all expressions about impacts concerned changes in daily performance and lifestyle for both responders (67.5%) and non-responders (63.2%) (**Supplementary Figure 1**). The most commonly reported impacts for both responders and non-responders were limitations to daily activities (20.7% and 21.7%, respectively), limitations to physical functioning (20.7% and 26.1%, respectively), and impacts to walking (20.7% and 21.7%). Additionally, anxiety or worry (20.7%) and the need to rest more (22.4%) were frequently reported impacts for responders. Approximately 15% of all expressions about impacts were about impacts on emotional health (17.7% for responders and 13.2% for non-responders).

At the EOT interviews, over two-thirds of all expressions about impacts concerned changes in daily performance and lifestyle for both responders (74.5%) and non-responders (66.7%), while over one quarter of non-responders reported decreased quality of life and/or increased treatment burden.

### 3.6 Treatment-related ocular symptoms

Thematic qualitative analysis was used to group ocular symptoms around the specific areas of interest, visual impairment, eye irritation, and eye pain. The severity ratings for these symptoms are reported in **Table 5**. By the C4 interviews, patients already had experienced improvements to their eye-related impacts. Nine patients reported an improvement to at least one of their ocular impacts at their C4 interview (**Table 6**). By the EOT interview, there was a substantial decrease in the mean severity of all 3 types of ocular symptoms, from their worst point during the study to the 2 weeks prior to the interview. The mean ratings for severity of eye pain decreased from 7.1 to 1.4, eye irritation decreased from 6.9 to 2.4, and visual impairment decreased from 8.0 to 3.5. Severity ratings over the 2 weeks preceding the EOT interview were at their lowest levels. Patients reported coping strategies to alleviate ocular symptoms that included applying eye drops and wearing sunglasses.

**Table 5:** Severity and bother of ocular symptoms.

Symptom*	At C4 Interviews (n=104)			At EOT Interviews (n=38)			
	Patients rating symptom, n (%)	Bother of symptom, mean (SD)	Severity rating for symptom “at worst”^†^ mean (SD)	Patients rating symptom, n (%)	Bother of symptom, mean (SD)	Severity rating for symptom “at worst”^†^ mean (SD)	Severity over past 2 weeks, mean (SD)
**Visual impairment^‡^**	59 (56.7)	6.6 (2.8)	6.7 (2.5)	27 (71.1)	7.3 (2.2)	8.0 (2.0)	3.5 (2.7)
**Eye irritation^¶^**	42 (40.4)	4.7 (2.4)	5.9 (2.2)	19 (50.0)	5.4 (2.9)	6.9 (2.6)	2.4 (2.5)
**Eye pain^§^**	12 (11.5)	6.1 (1.8)	6.6 (2.2)	12 (31.6)	6.8 (1.7)	7.1 (1.6)	1.4 (1.9)

*Disease and treatment-related symptom severity and bother were rated 0–10 with 10 representing maximum severity, respectively; ^†^Patients were asked to rate the severity of the symptom they thought was the worst for them during their time on the trial; ^‡^Includes poor vision, blurred vision, and sensitivity to light; ^¶^Includes irritated eyes, dry eyes, itchy eyes, and feeling that something is in the eye; ^§^Includes painful eyes, sore eyes, and burning.

C4, cycle 4; EOT, end of treatment; SD, standard deviation.

**Table 6:** Improvement in ocular symptoms.

Eye-related impact that had improved by C4 interview	Number of patients reporting each type of improvement	Example quotes
Decreased independence	1	*I drive a little bit at a time now just so I don’t have to depend on people.*
Difficulty driving	7	*Oh yes [I’m back to driving]. This was just a period of maybe three weeks here [eye dryness prevented driving].* *It has been maybe two, three months and the last two weeks ago, I felt like my eyes, I could see the street signs again.*
Difficulty on computer	1	*[The impact of eye dryness] is a lot better … [the impact is still there] slightly but hardly noticeable.*
Difficulty on phone/texting	1	*But I stopped using a magnifying glass to look at my phone, [laughs] that’s pretty small letters though.*
Difficulty reading	4	*I saw everything double, but now it’s improved substantially, and now I can read better again.* *I can read a book again.*
Difficulty watching television	1	*[The impact of eye dryness] is a lot better … [the impact is still there] slightly but hardly noticeable.*
Need for corrective lenses	1	*He didn’t know what happened, so I changed eyeglasses [back to the original prescription as my eyes were better].*
Eye-related impact that had improved by EOT interview		
Limitations to daily activities	2	*Not anymore, but in the beginning, [eye itchiness and burning] did really limit what I did during the day.* *Now not at all [dryness doesn’t affect day].*
Limitations to housework or chores	1	*I’ll do that myself. I can maintain that and now that my eyes are better, it’s a little easier, easier to cook.*
Difficulty driving	2	*I’ll drive anywhere from 5 to 10 miles [wasn’t driving before].* *I can drive legally now. I am able to drive now.*
Difficulty reading	3	*I can read, I have 20/20 and could not see with all that medicine … then get off of it and I can see really well.* *I just recently tried to start reading books again.* *I can read signs almost two blocks away now.*
Difficulty watching television	1	*It’s getting better now [being able to see the television].*

C4, cycle 4; EOT, end of treatment.

### 3.7 Treatment satisfaction

**Figure 1:**
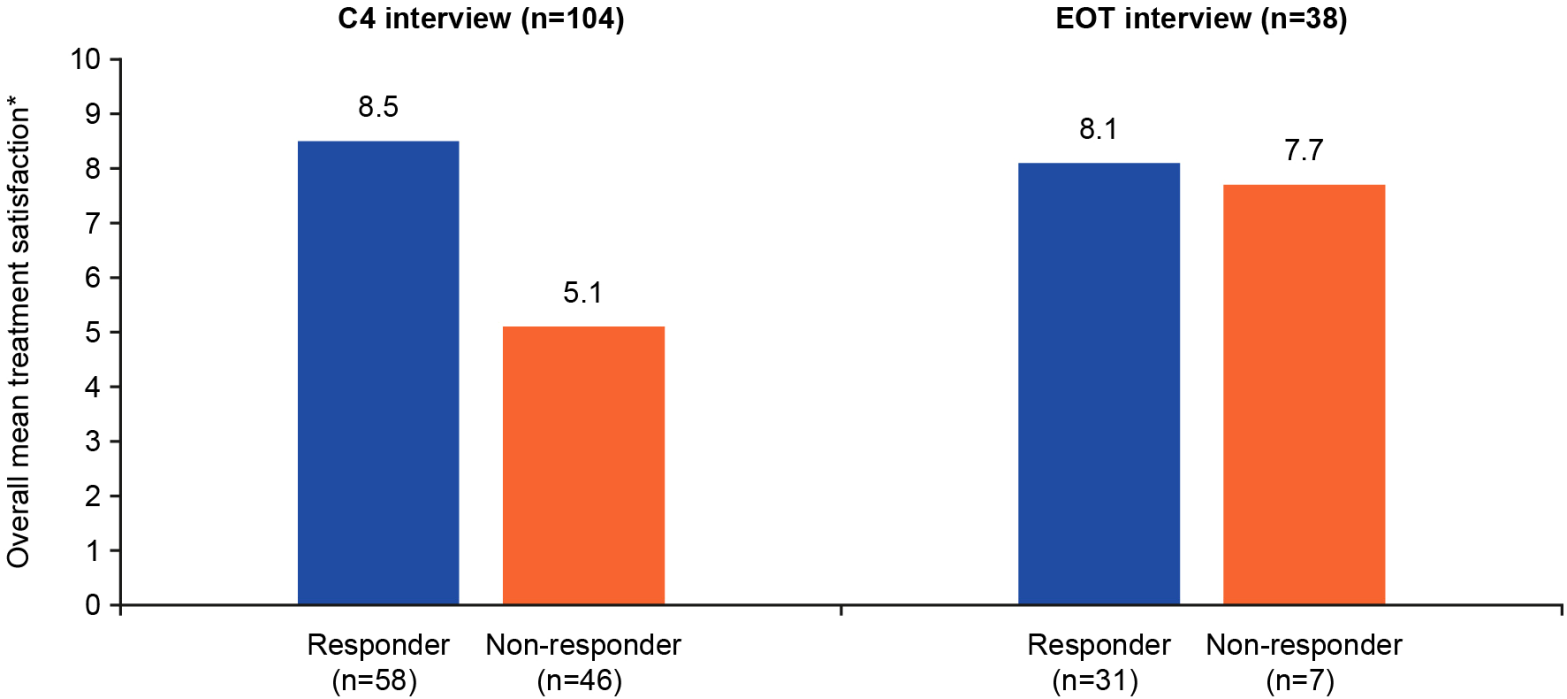
Quantitative summary of patient treatment satisfaction*. *Overall treatment satisfaction was rated 0–10 (0=not at all satisfied; 10=extremely satisfied). C4, cycle 4; EOT, end of treatment.

Patient satisfaction with treatment was rated on an NRS of 0–10, where 0=not at all satisfied and 10=extremely satisfied. Responders reported a higher satisfaction with treatment at both C4 and EOT interviews (8.5 and 8.1, respectively) than non-responders (5.1 and 7.7, respectively) (**Figure 1**). Examples of patients’ expressions of high, moderate, and low treatment satisfaction are presented in **Supplementary Figure 2**. Responders often reported being highly satisfied because the treatment was working for them, they felt better in general, or had improved symptoms. Ocular symptoms were discussed and considered when rating patients’ satisfaction. Patients reported being happy with their medical teams and feeling hopeful about the treatment’s continued effects in the future. Patients who were dissatisfied with the study treatment referred to the lack of treatment effect and the severity of the ocular symptom they developed during the study. Several patients were upset that they had been medically discontinued from the trial and had wanted to receive more treatments. However, other patients reported a positive experience in the trial and were satisfied with the care they received from their medical teams.

Patients’ quotes relating to their experience of the DREAMM-2 study are presented in **Supplementary Figure 3**. Patient responses were categorized as describing the treatment as easy (60.6% of all statements), difficult (28.7%), or neither easy nor difficult (10.6%). Descriptions of what made the treatment easy focused on drug administration being short in duration and not a bother, and some patients liked that the treatment was only given every 3 weeks, as opposed to every week. Descriptions of what made the treatment difficult focused on side effects of the study treatment, other procedures involved in study participation (such as laboratory tests and examinations), travel time to the clinic, and wait time at the clinic. Patient quotes encapsulating their perspectives on the risks and benefits of belantamab mafodotin treatment, which underline their acceptance of potential side effects as a necessary trade-off for improved health outcomes, are presented in **Supplementary Figure 4**. For patients with triple-class refractory RRMM, alternative treatment options are limited; therefore, the side effects experienced in DREAMM-2 were considered tolerable due to their temporary nature and lower severity compared with side effects experienced with other treatments.

## 4 Discussion

This embedded qualitative interview study explored the experiences of patients with RRMM who received treatment in the DREAMM-2 study. Results describe patients’ symptom experience at baseline, during treatment, and at EOT with belantamab mafodotin and how these symptoms impacted HRQoL. The majority of patients were responders to belantamab mafodotin treatment at their C4 interview. This is reflected in the higher overall response rate reported in interviewees (56.8% [n=63/111]) compared with those in the main DREAMM-2 population (32.0% [n=31/97] in the 2.5 mg/kg dose group; 35.4% [n=35/99] in the 3.4 mg/kg dose group and 52.0% (n=13/25) in the lyophilized cohort) (17, 21). Both belantamab mafodotin responders and non-responders experienced symptomatic benefit between study start and C4 treatment. Symptom severity improvement was greatest in patients who responded to belantamab mafodotin; however, non-responders also may have experienced symptom improvement but did not meet thresholds to be considered a responder.

Improvements in severity ratings from study start to C4 in treatment responders were observed for symptoms associated with RRMM including bone pain, bone fractures, numbness, and constipation. Improvements were also seen in fatigue, neuropathy, and back pain. In another study, He et al. observed that patients with newly-diagnosed MM and patients with RRMM cited bone pain and fatigue as the most disruptive to their HRQoL; improvements in these 2 categories were also among the most discussed treatment benefits (11). Similar observations were reported by Crawford et al. in their semi-structured interviews of patients with RRMM in which patients reported pain and fatigue as their most common symptoms (8).

With respect to ocular symptoms, the results indicate that symptoms associated with visual impairment, eye irritation, and eye pain were at their worst after C4 but were at- or near-resolution by the EOT interview. At the EOT interviews, patients reported improvements in ocular symptoms that had impacted on daily activities including reading, driving, using a computer/phone, and watching television. At C4 interview, only 4.8% of impact expressions were related to eye-related impacts. In an analysis of data from the DREAMM-2 study specific to corneal epithelial findings, Farooq et al. estimated that the majority of patients (80%) would recover from their ocular symptoms in approximately 2 to 6 months (20). The results from this analysis lend further support that the patient experience of ocular symptoms with belantamab mafodotin is mostly temporary and does not appear to have a long-term impact on overall quality of life in patients with triple-class refractory RRMM and exposed to 3 or more prior lines of therapy.

The interviews revealed a range of patient experiences, including positive, negative, and neutral perspectives on their treatment with belantamab mafodotin. The patient satisfaction ratings observed in this study are consistent with previous reports that emphasize the importance of HRQoL in patients with RRMM. Previous evaluations of patient-reported outcomes in RRMM populations have established that the disease burden of RRMM is high, and potentially higher than other advanced cancers (10). Larocca et al. showed in their analysis of patient-reported outcomes data from the HORIZON study that patients with RRMM reported a higher severity rating for pain than patients with any other type of cancer, assessed by the EuroQoL visual analogue scale (EQ VAS) (10). Parsons et al. found that fatigue was a highly important symptom to almost all patients interviewed, all of whom had RRMM. In this same study, patients ranked improvement in their physical wellbeing (e.g., fatigue) as a high priority, while predictable treatment-related adverse events were ranked as a lower priority (24). As pain and fatigue are strongly linked to poorer HRQoL, improvements in these symptoms are clinically important to patients, especially in later stages of disease as their symptomatic burden increases (10).

This embedded qualitative patient interview study provides valuable insight into the experience of patients with RRMM receiving belantamab mafodotin treatment. The interviews themselves, by merit of having a structured set of open-ended questions and probes, are both broader and deeper in scope than established HRQoL questionnaires included in clinical trials. Open-ended questioning elicits spontaneous, unstructured testimonials from patients. Exploring such qualitative data provides a fuller picture of the impact of RRMM and its treatment using belantamab mafodotin, to add context to the growing volume of clinical trial data in this setting and provide meaningful insights for healthcare providers.

The study had several limitations. As with most qualitative research, the sample was small in some groups at certain interview time points, limiting the ability of subsamples to be statistically representative to their larger populations. In this study, the interview sample contained a higher proportion of responders than the larger clinical trial. Inclusion in the study was dependent upon patients agreeing to participate in at least one interview, introducing a potential self-selection bias. Patients were enrolled from centers around the globe, increasing the likelihood of capturing a diverse sample of patient views. However, in addition to fewer non-responders, there was a potential bias from the numbers of patients enrolled from the US, and underrepresentation of racial minorities compared with the real-world population. Patients from different cultures may report symptoms differently in interviews. Finally, given that patients were selected from a clinical trial population, the results may not be fully reflective of a real-world population of patients with RRMM.

## 5 Conclusion

This qualitative patient interview study provides valuable insight into patients’ symptomatic experience with belantamab mafodotin for the treatment of RRMM. These insights may help healthcare providers to better anticipate their patients’ real-world experience and needs when prescribing this novel agent in clinical practice settings. The patients interviewed (particularly responders) were generally satisfied with treatment and reported improvements in disease symptoms. This evidence base should assist healthcare providers in tailoring clinical practices to understand, anticipate, and manage patients’ symptomatic experience while receiving belantamab mafodotin.

## Data availability statement

Information about GSK’s data sharing commitments and access requests to anonymized individual participant data and associated documents can be requested for further research from https://www.gsk-studyregister.com/en/.

## Ethics statement

The studies involving humans were approved by Institutional review board/ethics committees approvals for the interview study obtained by amendment to the main BMA 205678 clinical trial protocol. This study was conducted in accordance with the guidelines on good pharmacovigilance practices (European Medicines Agency, 2014), preference-based methods from International Society for Pharmacoeconomics and Outcomes Research (ISPOR), Good Practices for Outcomes Research (International Council for Harmonisation of Technical Requirements for Pharmaceuticals for Human Use, 1996), and applicable regulatory and country-specific requirements. The studies were conducted in accordance with the local legislation and institutional requirements. The participants provided their written informed consent to participate in this study.

## Author contributions

AC: Conceptualization, Data curation, Formal Analysis, Writing – review & editing. JC: Conceptualization, Data curation, Formal Analysis, Writing – review & editing. MM: Conceptualization, Data curation, Formal Analysis, Writing – review & editing. BG: Formal Analysis, Writing – review & editing. SS: Formal Analysis, Writing – review & editing. RP: Data curation, Formal Analysis, Writing – review & editing.
