# Supplementary material for: Patient-reported experiences during and following treatment with belantamab mafodotin for relapsed/refractory multiple myeloma in the DREAMM-2 study
Source: Front Oncol. 2023 Dec 7;13:1274659. doi: 10.3389/fonc.2023.1274659 (PMC10748584; doi:10.3389/fonc.2023.1274659)
Supplement: Supplementary file 1 [file DataSheet_1.pdf]

Supplementary Figure 1. Summary of impact concept code frequency at A) C4 and B) EOT interviews

(A)

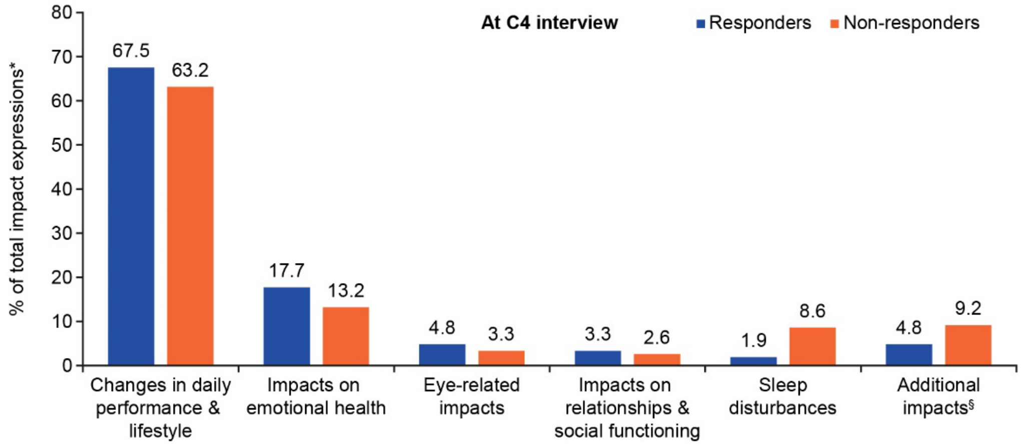

	Changes in daily performance & lifestyle		Impacts on emotional health		Eye-related impacts		Impacts on relationships & social functioning		Sleep disturbances		Additional impacts§	
Patient expressions†, n	141	96	37	20	10	5	7	4	4	13	10	14
Contributing transcripts‡, n (%)	31 (53.4)	25 (54.3)	18 (31.0)	9 (19.6)	2 (3.4)	1 (2.2)	5 (8.6)	3 (6.5)	3 (5.2)	5 (10.9)	6 (10.3)	8 (17.4)

(B)

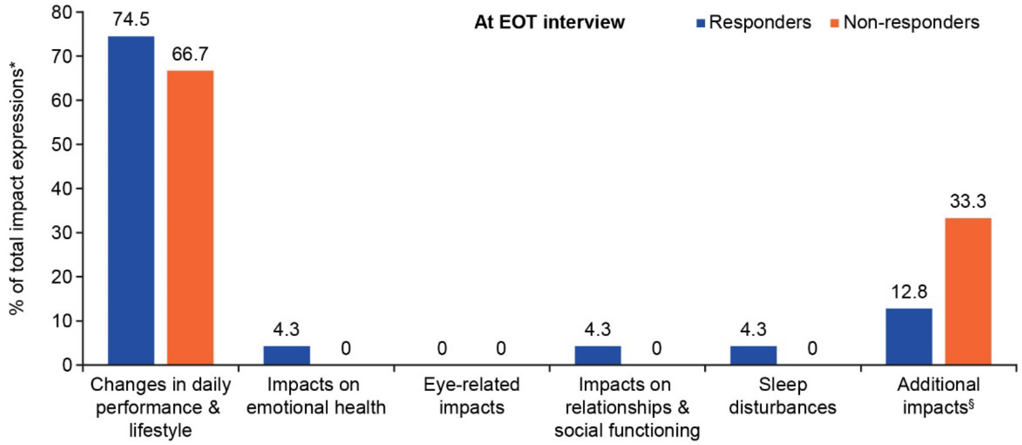

	Changes in daily performance & lifestyle		Impacts on emotional health		Eye-related impacts		Impacts on relationships & social functioning		Sleep disturbances		Additional impacts§	
Patient expressions†, n	35	6	2	0	0	0	2	0	2	0	6	3
Contributing transcripts‡, n (%)	17 (54.8)	2 (28.6)	2 (6.5)	0	0	0	1 (3.2)	0	1 (3.2)	0	3 (9.7)	2 (28.6)

*Total impact expressions: C4 (n=209 in responders, n=152 in non-responders) and EOT (n=47 in responders, n=9 in non-responders); †Number of patient language expressions with concept; ‡Number of transcripts contributing to concept expression: C4 (n=58 in responders, n=46 in non-responders) and EOT (n=31 in responders, n=7 in non-responders); §Includes decreased independence, decreased quality of life, economic burden, and treatment burden.

C4, cycle 4; EOT, end of treatment.

Supplementary Figure 2. Patient quotations about satisfaction with belantamab mafodotin treatment during the DREAMM-2 study

High Treatment Satisfaction

"I was 100% satisfied. If I had to take the eye thing into concern, I would say I was 80% satisfied."

"So far this treatment has worked as well or better than the ones I've had over the years. I would say being ten plus years into this, I'm 90% satisfied."

"100% [satisfied with the treatment]. *Because it's worked so well and so quickly. Even after the first cycle, the system they used to measure the myeloma, which is the light chain figure, it went down to zero straight away. I mean, like you said, something about how successful the trial drug is...10"*

"I'm tickled to death with what the treatment has done to my cancer. I mean it's dropped my levels tremendously..."

Moderate Treatment Satisfaction

"So far I'm satisfied because the doctors are doing everything, they can... and I'm hoping for the best... not knowing one way or the other we'd have to go with a five then."

"I'd give it a 7 or 8 or something like that. I mean it didn't work for me, but it might work for other people. Just because it didn't work for me don't mean it's [not] *going to work for somebody else."*

"I'd like to say right in the middle of the road. If it weren't for the eyes, I'd be right up there with a 10, but with the eyes, you know, your eyesight, how much value you put on that, I'd say a 6 or so. It's a great drug for me."

"Moderately [satisfied]. *That's kind of like a C... I'm happy that the myeloma numbers went down somewhat. I don't know if discouraged would be the right word but dealing with some of the side effects... a 5... middle of the road.."*

Low Treatment Satisfaction

"I would get in remission, so I would be real happy... and if not, just keep me where I'm at, I'd be happy, yeah."

"Well, if it's not effective, I wouldn't be satisfied...it was not effective so I can't say I'm satisfied with it, right... Right now I have to give it zero because there's no improvement in my condition."

Supplementary Figure 3. Patient quotations regarding their patient experience during the DREAMM-2 study

Study Treatment Was Easy	Study Treatment Was Difficult	Study Treatment Was Neither Easy Nor Difficult (or Mixed)
“Oh, it's easy. The treatment itself is half an hour in a hospital chair. It's really no problem at all. It's brilliant...”	*“I had to take off every three weeks from work, you know, everyone’s going on like week-long vacations and I can’t do that because I need to save my time off for this..”*	*“Difficult, easy…. everything was very normal like in any other chemotherapy.”*
“Apart from everything they need, the information that they need on the trial, the actual infusion which is just an infusion is very, very straightforward.”	*“... there were often times when I had to wait for the medication to be prepared for as much as two hours even three hours at one time. And the information didn’t always seem to flow between the cohorts as well as I felt it should have.”*	*“It's a little difficult because I got joint pains, but then easier because I go home every night* [compared to other treatments I've had]*.”*
“It was fairly easy, I mean it’s pretty regular in terms of having to go to the eye doctor. But they did it all in one day.”	*“I travel to go to my hospital and to travel all that way and then have it postponed that was frustrating.”*	
“Actually, taking the treatment is relatively easy so all of that has been not a problem.”	*“Well, the most irritating thing is your eye exam.”*	
“What makes it easy for me is that I feel well.”	*“The first one was a bit hard because of the fever.”*	

Supplementary Figure 4. Patient quotations about risks and benefits of belantamab mafodotin treatment during the DREAMM-2 study

Staying on Study Treatment Despite New Symptoms

“I make the call usually to push ahead because I’m out of options now. I’ve been through every standard treatment. So, it would only be trials, that’s all I have available..”

“I thought seriously about not continuing, and if there had been another drug for me at this point, approved and ready for me to get it, I might have stopped it, but there is not...so I just decided that I would just put up with not being able to see.”

“No [never thought of stopping], *knowing* [side effects] *were temporary, they come and go is not bothering – I’m more about staying alive. Except for my eyes I do feel better...so I’m all for it.”*

“It is working on the cancer right now so it seems to be a tradeoff... take the drug...put up with blurry vision and reassess down the road.”

“So far, the thing that only happens is the blurry eyes, that’s all...No [I didn’t think about stopping]. *It helps my pain...it works pretty good on my myeloma.”*

Weighing Risks & Benefits

“Aside from the eyes I had no other side effects, and I don’t know if the eyes will recur or if they won’t but even if they do, that’s okay. I’d rather be alive.”

“There are side effects to absolutely any treatment you have and some of the treatments I’ve had have had much worse than this.”

“It was either that [side effects] *or go through the possibility of...light chains.”*
